# Global development of bispecific antibodies in oncology: a bibliometric analysis of geographic disparities, collaborative models, and emerging translational strategies

**DOI:** 10.3389/fimmu.2026.1884087

**Published:** 2026-08-20

**Authors:** Xiaotian Zhai, Zhuona Rong, Lixia Fu, Yanlun Gu, Bingqi Dong, Dongning Yao, Xiaocong Pang, Yimin Cui

**Affiliations:** 1 Institute of Clinical Pharmacology, Peking University First Hospital, Beijing, China; 2 Beijing Key Laboratory of Clinical Pharmacology and Translation of Innovative Drugs, Beijing, China; 3 School of Pharmaceutical Sciences, Peking University, Beijing, China; 4 Department of Pharmacy, Peking University First Hospital, Beijing, China; 5 Department of General Surgery, Peking University First Hospital, Beijing, China; 6 School of Pharmacy, Nanjing Medical University, Nanjing, China

**Keywords:** adverse event, bispecific antibody, blinatumomab, clinical trial, collaboration network

## Abstract

**Background:**

Bispecific antibodies (bsAbs) have rapidly emerged as one of the most promising therapeutic strategies in oncology because of their ability to simultaneously engage multiple molecular targets and enhance antitumor immunity. Although the number of publications has increased substantially, a comprehensive bibliometric characterization of the global research landscape and its developmental trajectory remain lacking.

**Methods:**

A bibliometric analysis was conducted using publications retrieved from the Web of Science Core Collection and Scopus from January 2014 to July 2026. After data standardization and deduplication, 5,929 eligible publications were included. CiteSpace, VOSviewer, and Bibliometrix were applied to evaluate publication trends, collaborations among countries, institutions, and authors, influential journals, keyword co-occurrence, citation bursts, and thematic evolution.

**Results:**

Global publications on bsAbs demonstrated sustained growth, particularly after 2018, reflecting rapidly increasing scientific interest. The USA and China contributed the largest research outputs, while several internationally collaborative institutions served as major research hubs. Highly cited journals and influential authors formed interconnected knowledge networks that characterized the scholarly structure of the field. Keyword clustering and timeline analyses revealed a progressive transition from antibody engineering and T-cell engager technologies toward clinical applications involving hematologic malignancies, solid tumors, combination immunotherapy, tumor microenvironment, and emerging therapeutic targets. Citation burst analyses further identified evolving research hotspots and highlighted increasing attention to translational and clinically oriented studies.

**Conclusion:**

This study reconstructs the global intellectual landscape and thematic evolution of oncology-oriented bsAb research. The findings provide a bibliometric overview of scientific development, identify current research frontiers, and may inform future collaboration, research prioritization, and investigation of next-generation bsAb therapies.

## 1 Introduction

Bispecific antibodies (bsAbs) have emerged as an important class of engineered immunotherapies capable of simultaneously engaging two distinct molecular targets, thereby enhancing tumor specificity and immune activation compared with conventional monoclonal antibodies (1–4). While monoclonal antibodies revolutionized oncology through target-specific inhibition, their limitations in overcoming tumor heterogeneity and immunosuppressive tumor microenvironments have catalyzed the rise of bsAbs (3, 5–7). The clinical success of blinatumomab, a bispecific T-cell engager targeting CD19 and CD3, marked a paradigm shift in relapsed or refractory B-cell malignancies; median overall survival was 7.7 months with blinatumomab versus 4.0 months with standard chemotherapy (8–11). To date, 18 bsAbs have received regulatory approval for cancer treatment, yet their therapeutic potential remains constrained by critical barriers, such as suboptimal response rates in solid tumors, dose-limiting toxicities including cytokine release syndrome, and a lack of validated biomarkers for precision patient stratification (1, 7, 12–14). The rapid expansion of bsAb-related publications reflects increasing scientific interest in antibody engineering, immune-cell redirection, and translational oncology; however, the overall evolution of this research field has not been comprehensively characterized.

The evolution of bsAbs has been propelled by iterative engineering breakthroughs. Early constructs grappled with manufacturing complexities and immunogenicity, but innovations like CrossMab technology and Dual-Affinity Re-Targeting (DART) platforms enabled scalable production of stable, IgG-like molecules (3, 4, 15–19). Recent key studies, including the approval of tarlatamab for small-cell lung cancer in 2024, underscore both progress and persisting challenges; the agent demonstrated a 25% objective response rate, and late-onset adverse events emerged in 34.8% of responders with mild cytopenia or asthenia after 1-year treatment (20–22). Despite the rapid expansion of mechanistic studies, engineering research, and clinical trials involving bsAbs (1, 3, 5, 7, 23), there has been no comprehensive bibliometric analysis that examines the developmental patterns of approved bsAbs, the distribution of research resources and research priorities needed to address current development bottlenecks, and accelerate the translation of novel bsAb candidates. Numerous reviews have summarized the engineering strategies, mechanisms of action, and clinical applications of bsAbs; these studies primarily focus on therapeutic advances rather than the evolution of the research landscape itself. Consequently, important questions remain unanswered regarding how scientific collaborations have evolved, which research themes have driven the field, and what emerging directions are shaping future oncology-oriented bsAb development.

To address this knowledge gap, we performed a comprehensive bibliometric analysis of global oncology-oriented bsAb research based on 5,929 publications retrieved from the Web of Science Core Collection (WoSCC) and Scopus. Using CiteSpace, VOSviewer, and Bibliometrix, we characterized publication trends, international collaborations, influential journals, institutions, authors, and thematic evolution. Citation burst detection, keyword clustering, timeline visualization, and thematic evolution analyses were employed to identify emerging research frontiers and shifting scientific interests. The findings provide a bibliometric knowledge framework that may facilitate future research prioritization, interdisciplinary collaboration, and strategic development of next-generation bsAb therapeutics.

## 2 Methods

### 2.1 Data sources

Bibliometric analysis is widely used to evaluate literature and map scientific fields by characterizing research development, knowledge structures, and emerging trends in specific fields (24). Performance analysis summarizes bibliographic characteristics, including publication output, countries, institutions, authors, journals, and keywords, whereas science mapping visualizes the relationships among these elements through network analysis (24). Co-citation analysis is a commonly applied bibliometric method to explore the intellectual structure of a research field by evaluating the frequency with which publications, authors, or journals are cited together.

Scopus and WoSCC were selected as the primary data sources for this bibliometric analysis owing to their complementary strengths in biomedical research evaluation (25, 26). Both are widely used multidisciplinary bibliographic databases that apply journal-selection criteria and provide standardized metadata exports, thereby supporting reproducible bibliometric analysis (26, 27). Specifically, WoSCC emphasizes selective, high-impact indexing in biological sciences and clinical research, whereas Scopus offers broader disciplinary inclusion, including engineering, pharmacy, and health policy (26, 28). The combination of these two databases was used to enhance literature coverage and reduce potential bias associated with using a single database.

### 2.2 Search strategy and data preprocessing

**Figure 1:**
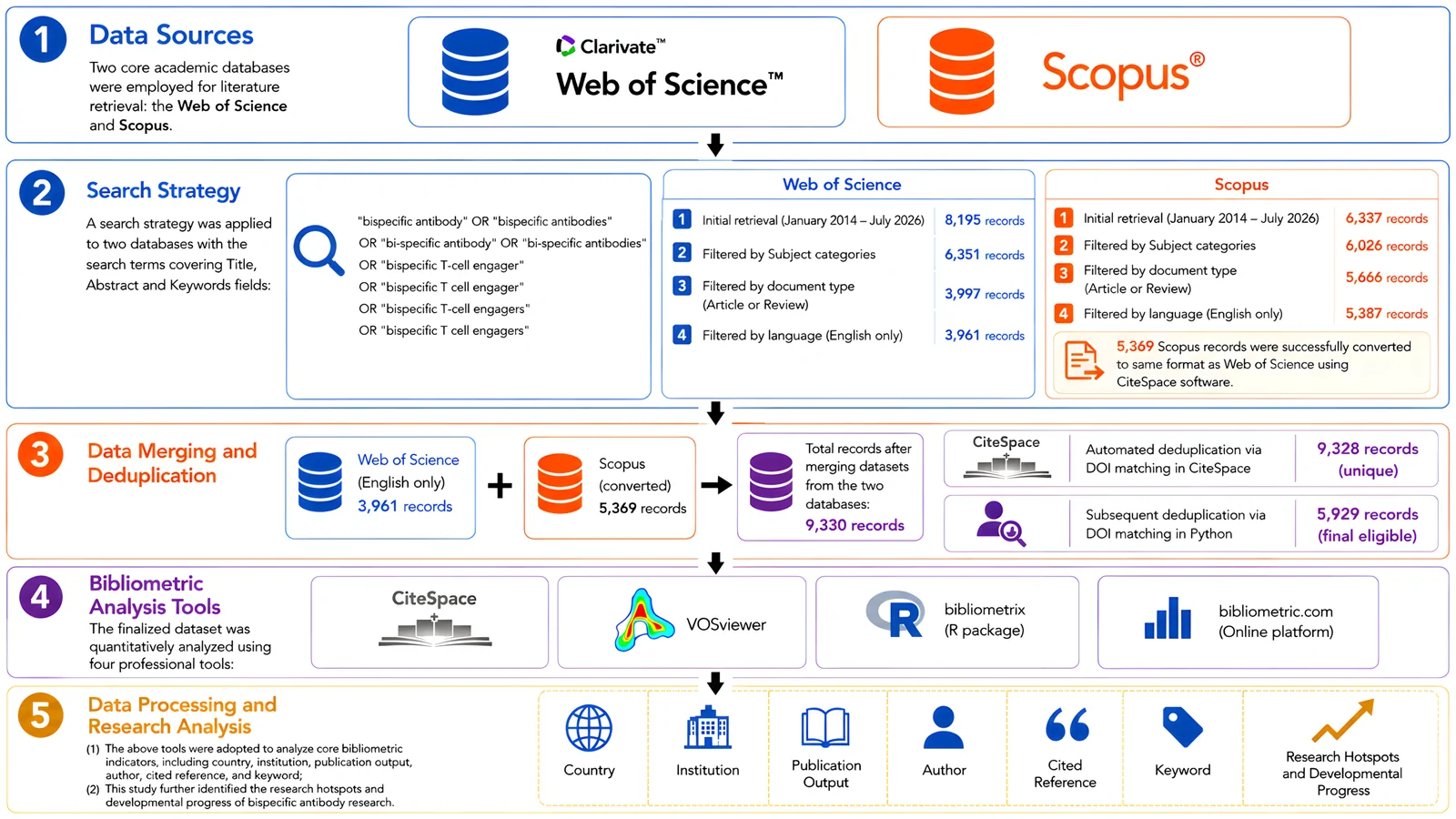
Flowchart of data screening and analysis.

The literature search was conducted to identify publications related to bsAbs. The search strategy was independently performed in WoSCC and Scopus using title, abstract, and keyword fields. The search terms were “bispecific antibody” OR “bispecific antibodies” OR “bi-specific antibody” OR “bi-specific antibodies” OR “bispecific T-cell engager“ OR “bispecific T cell engager” OR “bispecific T-cell engagers” OR “bispecific T cell engagers”. The records were retrieved within a time span of January 2014 to July 2026 (**Figure 1**). For WoSCC, publications were restricted to the following categories: Oncology; Hematology; Immunology; Pharmacology and Pharmacy; Medicine Research Experimental.

For Scopus, the following subject areas were selected: Medicine; Biochemistry, Genetics and Molecular Biology; Immunology and Microbiology; and Pharmacology, Toxicology and Pharmaceutics. Only English-language articles and reviews were included.

The retrieved records were screened based on titles and abstracts to exclude publications that were not directly related to bsAb research. Eligible records were exported with complete bibliographic information and cited references. To facilitate the analysis of records across different software platforms, Scopus records were converted into a WoSCC-compatible format using CiteSpace. Records retrieved from WoSCC and Scopus were subsequently merged. Duplicate records were initially identified and removed using DOI-based matching in CiteSpace. The merged dataset was subsequently screened using DOI information to identify and remove remaining duplicate records via Python (version 3.8). The final unique dataset was used for subsequent bibliometric analyses.

To improve the accuracy of collaboration analyses, names of authors, institutions, countries, and journals were manually standardized where necessary by correcting spelling variations, abbreviations, institutional mergers, and duplicate entities prior to visualization. Country names were standardized according to ISO 3166 country codes, with variant spellings and territorial designations merged. Institutional names were manually standardized for high-frequency entities. Author names were standardized with manual disambiguation for high-frequency authors with identical initials. Journal names were matched to official names to merge variant name formats. This study followed a structured bibliometric workflow for literature retrieval and quantitative knowledge mapping. The analytical workflow is illustrated in **Figure 1**, and all analyses were conducted using the same deduplicated bibliographic dataset to ensure consistency across software platforms.

### 2.3 Bibliometric and science-mapping analyses

Bibliometric analyses were conducted using VOSviewer (version 1.6.20), CiteSpace (version 7.0.R0 Advanced), and Bibliometrix (R version 4.5.1). VOSviewer was primarily used to visualize country-level citation networks and source co-citation relationships. CiteSpace was applied to identify collaboration networks, keyword co-occurrence, citation bursts, and temporal knowledge evolution. Bibliometrix was used to perform descriptive bibliometric analyses, including annual publication trends, Bradford’s law, Lotka’s law, thematic evolution, and three-field plots. Detailed parameter settings, thresholds, clustering algorithms, and normalization methods are provided in **Supplementary Table 3**.

## 3 Results

### 3.1 International collaboration network

**Figure 2:**
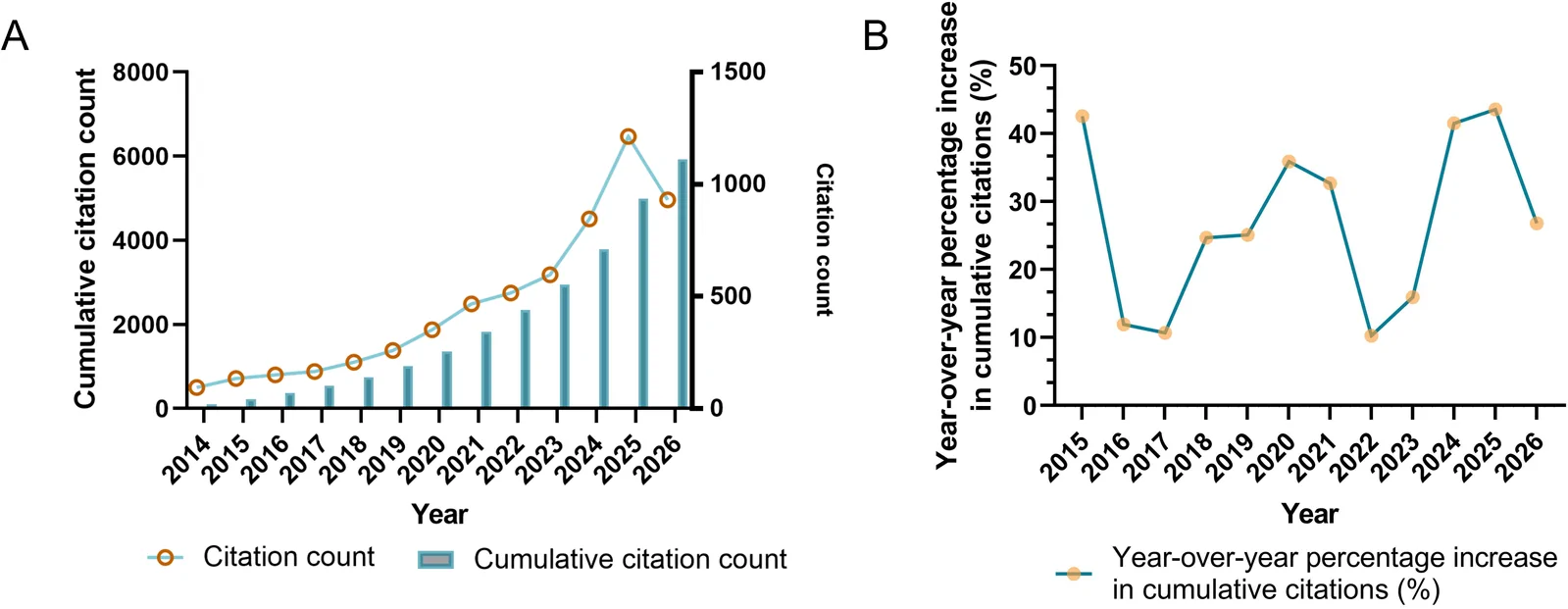
Annual citation trends in bsAb research (2014–July 2026). **(A)** Annual citation counts and cumulative citation counts. **(B)** Year-over-year percentage increase in cumulative citations. For each year *t*, the percentage increase was calculated as ([*C_t_* − *C_t_*_–1_]/*C_t_*_–1_) × 100%, where *C_t_* and *C_t–_*_1_ represent the cumulative citation counts at the end of year *t* and the preceding year, respectively. The metric therefore represents the percentage increase in cumulative citations relative to the immediately preceding year. No value was calculated for 2014 because no preceding-year cumulative citation count was available. The 2026 value was based on data available through July 2026 and should therefore be interpreted cautiously.

**Figure 3:**
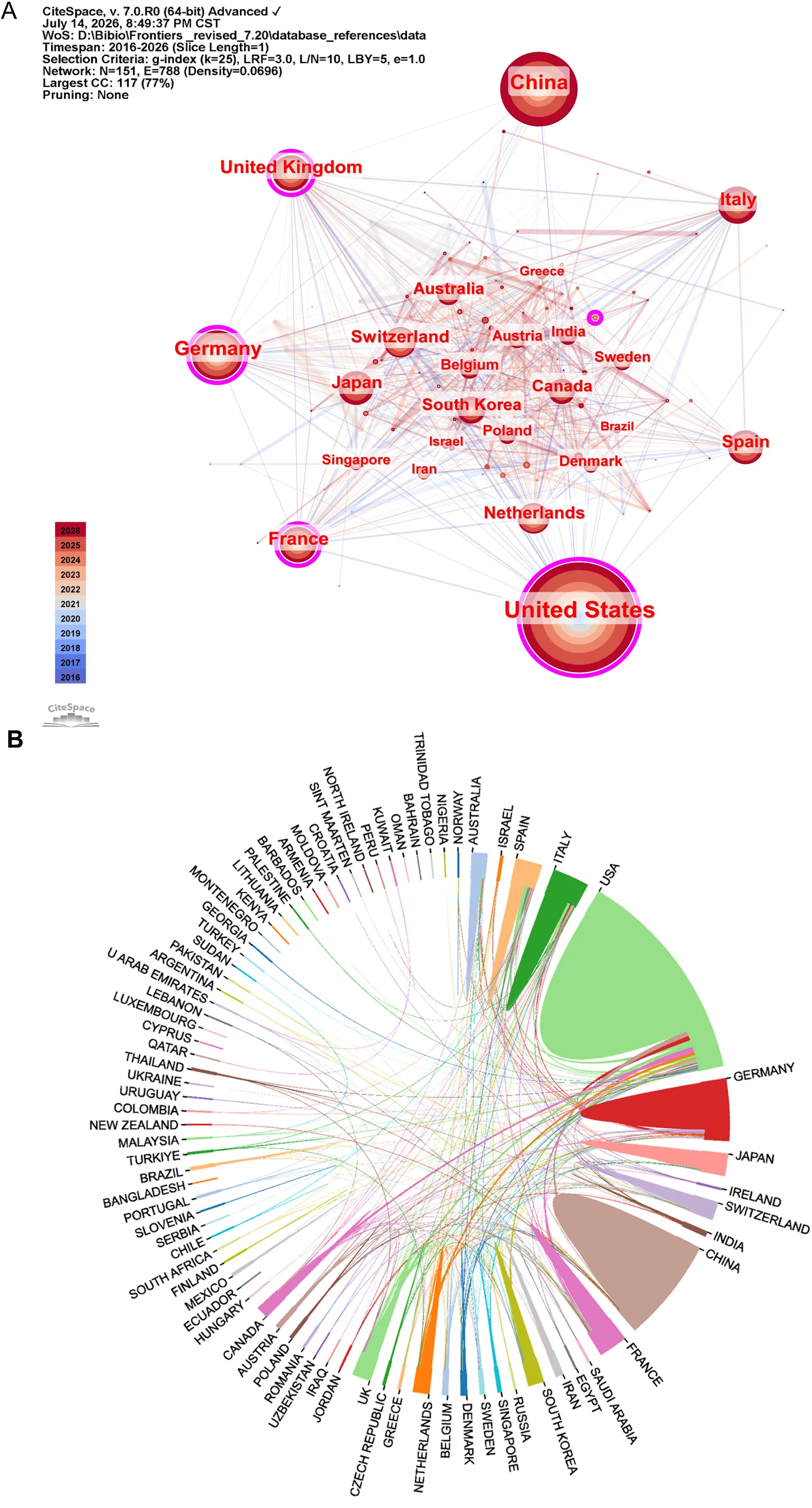
International collaboration network of countries involved in bsAb research. **(A)** Collaboration network of countries by CiteSpace. Node size represents publication frequency, and links indicate collaborative relationships between nodes. Node colors indicate the year in which each country first appeared in the collaboration network. Nodes with betweenness centrality greater than 0.10 are highlighted with purple outer rings. High betweenness centrality reflects the structural bridging role of a country within the collaboration network rather than research quality or clinical importance. **(B)** Collaboration network of countries by the online tool bibliometric.com. Different colored sectors indicate different countries, links indicate collaborations, and sector sizes indicate the document count of each country.

To characterize temporal trends in scholarly attention to bsAb research, we first analyzed annual and cumulative citation counts from 2014 to July 2026 (**Figure 2A**). Cumulative citations increased throughout the study period. The year-over-year percentage increase in cumulative citations varied considerably across years (**Figure 2B**), with a mean of 26.8% across the complete annual intervals analyzed. Because citation data for 2026 were available only through July, the 2026 value was not included in the calculation of the mean and was interpreted separately. The international collaboration network for the research and development of bsAbs over the past decade was visualized, and all literature sources were analyzed by CiteSpace and the online tool Bibliometrix (**Figure 3**). We observed that in the global bsAb literature, the USA, China, and Germany occupied prominent positions, indicating a central role in the international collaboration network. The USA exhibited the highest publication output (**Table 1**), the greatest citation count (**Table 1**), and the largest node size in the CiteSpace collaboration network (**Figure 3A**). Node size represents publication frequency, links indicate collaborative relationships, and purple rings denote high betweenness centrality (> 0.10), suggesting potential bridging roles. The USA, the UK, France, and Germany had high betweenness centrality and therefore occupied important bridging positions in the international bsAb collaboration network (**Figures 3A, B**). Overall, bibliometric analyses consistently demonstrated increasing publication and citation activity throughout the study period, while country-level analyses revealed marked heterogeneity in publication output, citation impact, and structural connectivity.

**Table 1:** Top 10 countries with a minimum of 90 documents (from VOSviewer).

Country	Document count	Citation count	Share of total citations (%)	Average citation per publication	Betweenness centrality
USA	2,620	93,148	38.79	35.55	0.8
China	1,219	25,712	10.71	21.09	0.02
Germany	711	32,841	13.67	46.19	0.69
France	346	16,240	6.76	46.94	0.7
Spain	316	16,077	6.69	50.88	0.37
Netherlands	277	13,741	5.72	49.61	0.32
UK	234	12,230	5.09	52.26	1
Switzerland	309	11,548	4.81	37.37	0.37
Italy	391	9,607	4.00	24.57	0.44
Canada	203	9,010	3.75	44.38	0.32

Share of total citations (%) = citation count/total citation count × 100.

Average citations per publication = citation count/publication frequency.

### 3.2 Global institutional collaboration network

**Figure 4:**
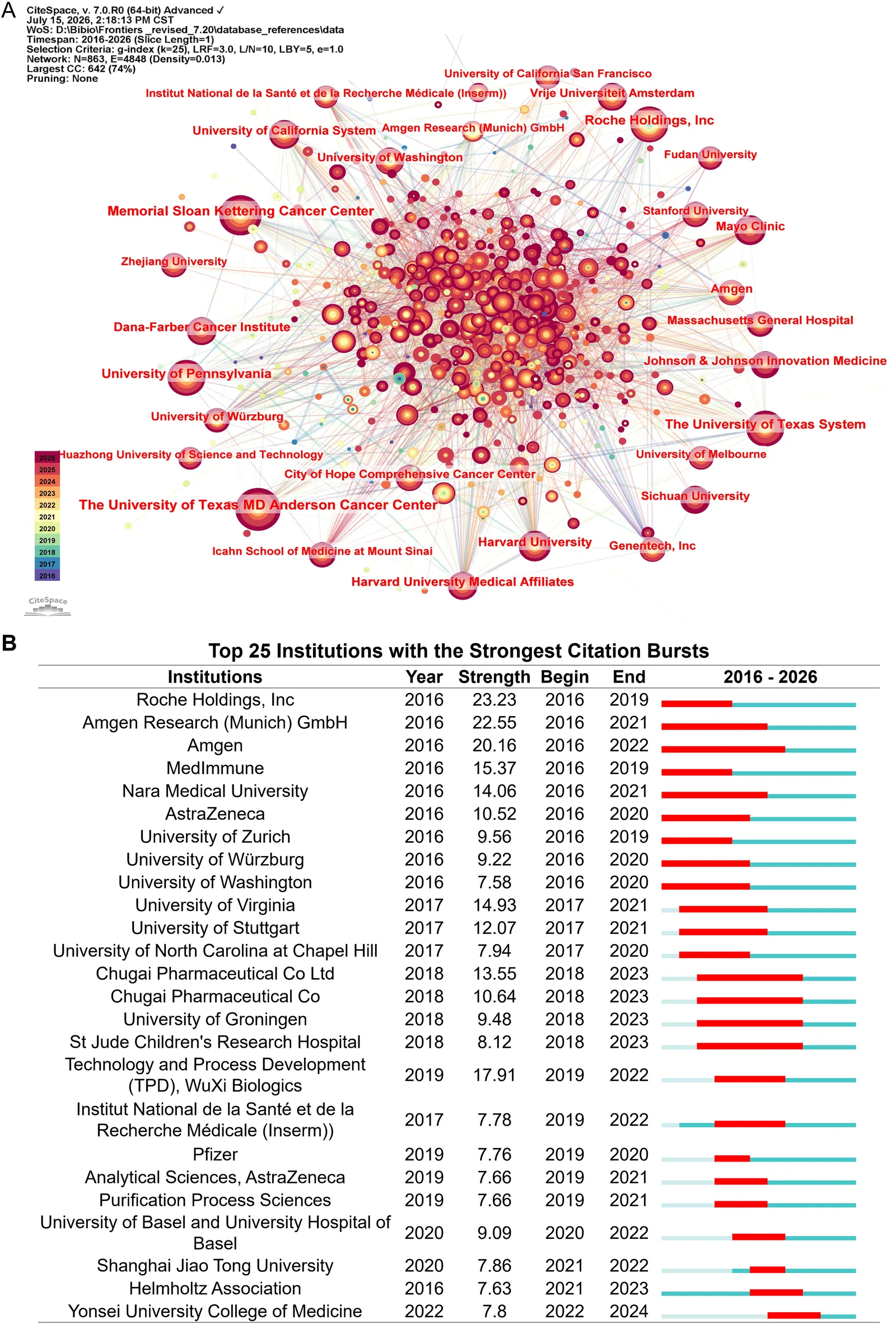
Institutional collaboration network and citation burst analysis in bsAb research. **(A)** Institutional collaboration network by CiteSpace. Each node represents an institution. Links represent collaborative relationships between institutions. Node size represents publication frequency. Node colors indicate the year in which each institution first appeared in the dataset. **(B)** Institutional burst detection by CiteSpace. Burst strength reflects the relative intensity of a sudden increase in citation attention during a specific time interval as calculated by CiteSpace. Institutions are ranked according to burst strength. The “year”, “strength”, “begin”, and “end” columns report the corresponding CiteSpace outputs, and the “2016–2026” column shows the overall detection period; red bars indicate burst duration. Burst detection identifies periods of rapidly increasing scholarly attention rather than research quality or clinical impact.

Institutional collaboration networks provide an overview of collaborative relationships and structural connectivity among research organizations, but do not establish whether these collaborations directly influenced translational or clinical development (29, 30). In this study, affiliations were extracted from all retrieved references using CiteSpace, generating 863 institutional nodes and 4,868 links, indicating an extensive collaborative network (**Figure 4A**). The network included universities, hospitals, research institutes, and pharmaceutical companies, indicating that bsAb research has developed through broad multi-institutional cooperation.

Citation burst analysis was subsequently performed to identify institutions that experienced rapidly increasing scholarly attention during specific periods. We used CiteSpace burst detection to analyze the citation dynamics of publications from these institutions and found 25 institutions with the strongest citation bursts, indicating rapidly increasing citation attention during specific periods (**Figure 4B**). Early burst institutions were primarily pharmaceutical companies, whereas more recent burst institutions increasingly included academic medical centers, reflecting evolving research participation over time. The collaboration network revealed extensive institutional interactions across academia, hospitals, and industry. Burst analysis further identified institutions that attracted rapidly increasing academic attention during different periods. Together, these complementary analyses indicate differences in institutional connectivity and temporal citation dynamics within the bsAb research landscape.

### 3.3 Publication network analysis

**Figure 5:**
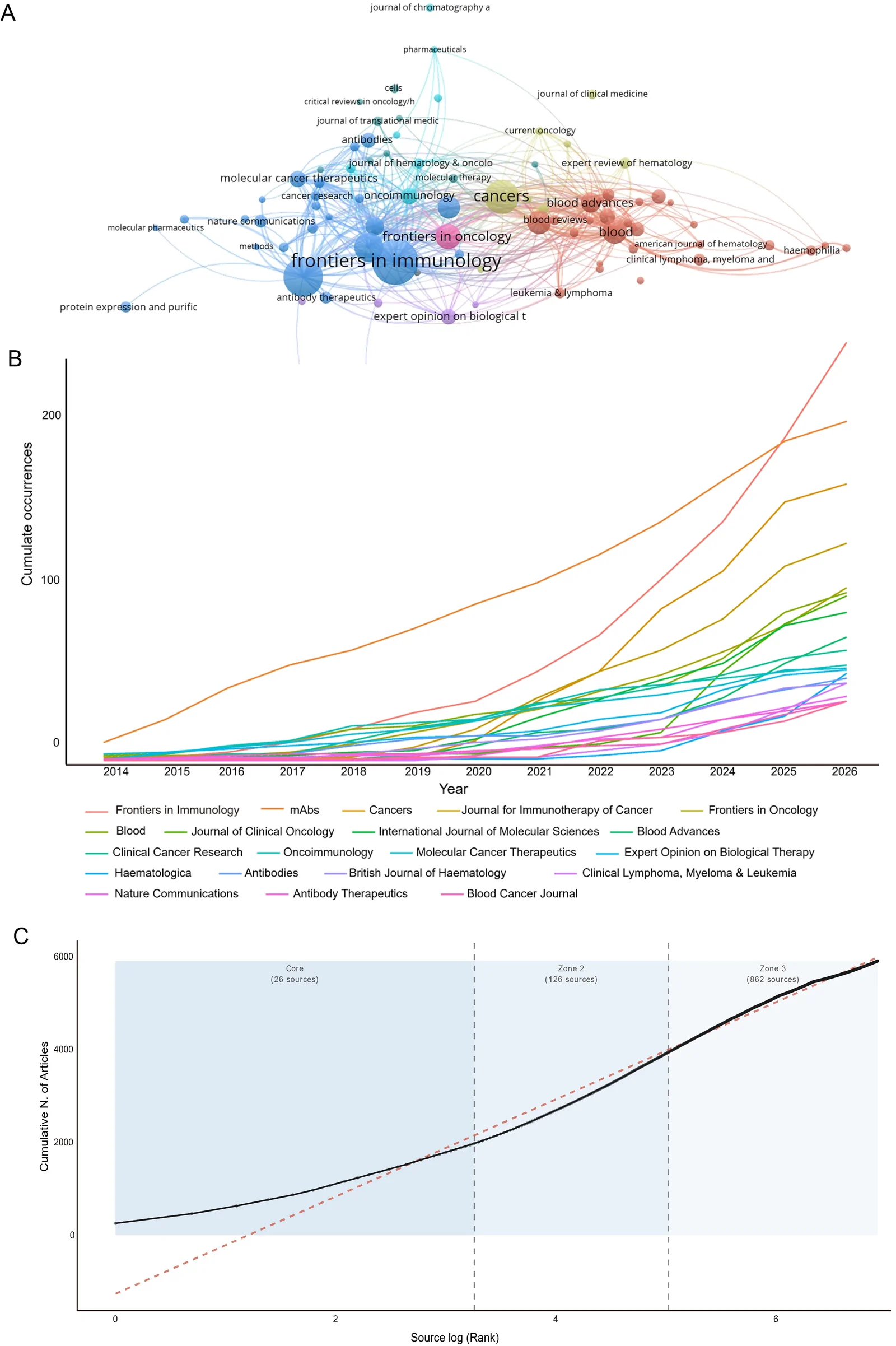
Publication landscape and journal distribution of bsAb research. **(A)** Journal co-citation network generated using VOSviewer. The analysis was performed in VOSviewer using cited sources as the unit of analysis, full counting, and association-strength normalization. Node size represents citation frequency, links indicate that two sources were cited together, and link thickness reflects co-citation strength. Colors represent clusters of closely related cited sources, and shorter distances indicate stronger normalized relatedness. Clusters were identified by the VOSviewer clustering algorithm. **(B)** Temporal publication trends of the 20 most productive journals generated using the R package Bibliometrix. **(C)** Bradford’s Law distribution of journals generated using the R package Bibliometrix.

To characterize the publication landscape of bsAb research, journals contributing to the retrieved literature were analyzed using co-citation network analysis and Bradford’s Law. We analyzed all the journals in which the retrieved bsAb literature was published. The co-citation analysis identified four major clusters (**Figure 5A**), representing closely related research communities in immunology, hematology, oncology, and translational medicine. The largest cluster centered on immunology-related journals, including Frontiers in Immunology. Science Translational Medicine (average citation = 123.00), Journal of Hematology & Oncology (average citation = 90.78), and Blood (average citation = 85.41) had the highest average citation counts per publication. These journals exhibited the highest average citations per publication (**Table 2**), indicating comparatively high citation visibility within the retrieved dataset. Among the leading journals, Frontiers in Immunology, mAbs, and Cancers exhibited the greatest increase in cumulative publication counts during the study period, whereas publication output in several traditional journals increased more gradually (**Figure 5B**).

**Table 2:** Top 10 journals with a minimum of 15 documents (from VOSviewer).

Rank	Journal	IF (2025)	JCR	Publications	Total citations	Average citations per publication
1	Blood	23.9	Q1	102	8,712	85.41
2	Mabs	7.9	Q1	206	6,647	32.27
3	Frontiers in Immunology	7	Q1	254	6,562	25.83
4	Journal of Clinical Oncology	44.7	Q1	100	4,572	45.72
5	Clinical Cancer Research	10.9	Q1	67	3,757	56.07
6	Journal for Immunotherapy of Cancer	11.7	Q1	132	3,614	27.38
7	Science Translational Medicine	15.6	Q1	29	3,567	123.00
8	Cancers	4.8	Q2	168	2,947	17.54
9	Journal of Hematology & Oncology	47.8	Q1	54	4,902	90.78
10	Blood Advances	7.7	Q1	75	2,188	29.17

Average citations per publication = total citation count/publication count.

IF, impact factor; JCR, journal citation reports.

Bradford’s Law is a key principle in bibliometrics that describes the distribution of information across journals or sources in a particular field (31, 32). Blood showed high citation visibility despite a comparatively moderate annual publication output. Of the top 10 bsAb core journals, nine journals were in the JCR Q1 quartile (**Table 2**). Application of Bradford’s Law divided the retrieved journals into three productivity zones (**Figure 5C**). Zone 1 contained only 26 journals (2.6% of all journals) but contributed publication output comparable to that of the much larger Zone 2 and Zone 3 (**Table 3**). Journals in Zone 1 are listed in **Supplementary Table 1**. Although Zone 1 accounted for only 2.6% of all journals, it contributed a publication volume comparable to that of the other two Bradford zones, indicating a high concentration of publication activity within a limited number of core journals. The journal landscape of bsAb research was characterized by both citation concentration in a limited number of established journals and rapid publication growth in several specialized or open-access journals. Bradford’s Law further showed that a small core group of journals accounted for a substantial proportion of the retrieved literature. Together, these complementary analyses indicate that bsAb research is disseminated through a relatively small group of highly productive core journals while publication activity has expanded across a broader range of specialized journals in recent years.

**Table 3:** All journals classified into three zones by Bradford’s Law using the R package Bibliometrix.

Zone	Journal number	Publications	Percentage of journals (%)
1	26	1,971	2.6
2	126	1,970	12.4
3	862	1,962	85

Percentage of journals (%) = journal number/sum of journal numbers × 100.

### 3.4 Author collaboration network

**Figure 6:**
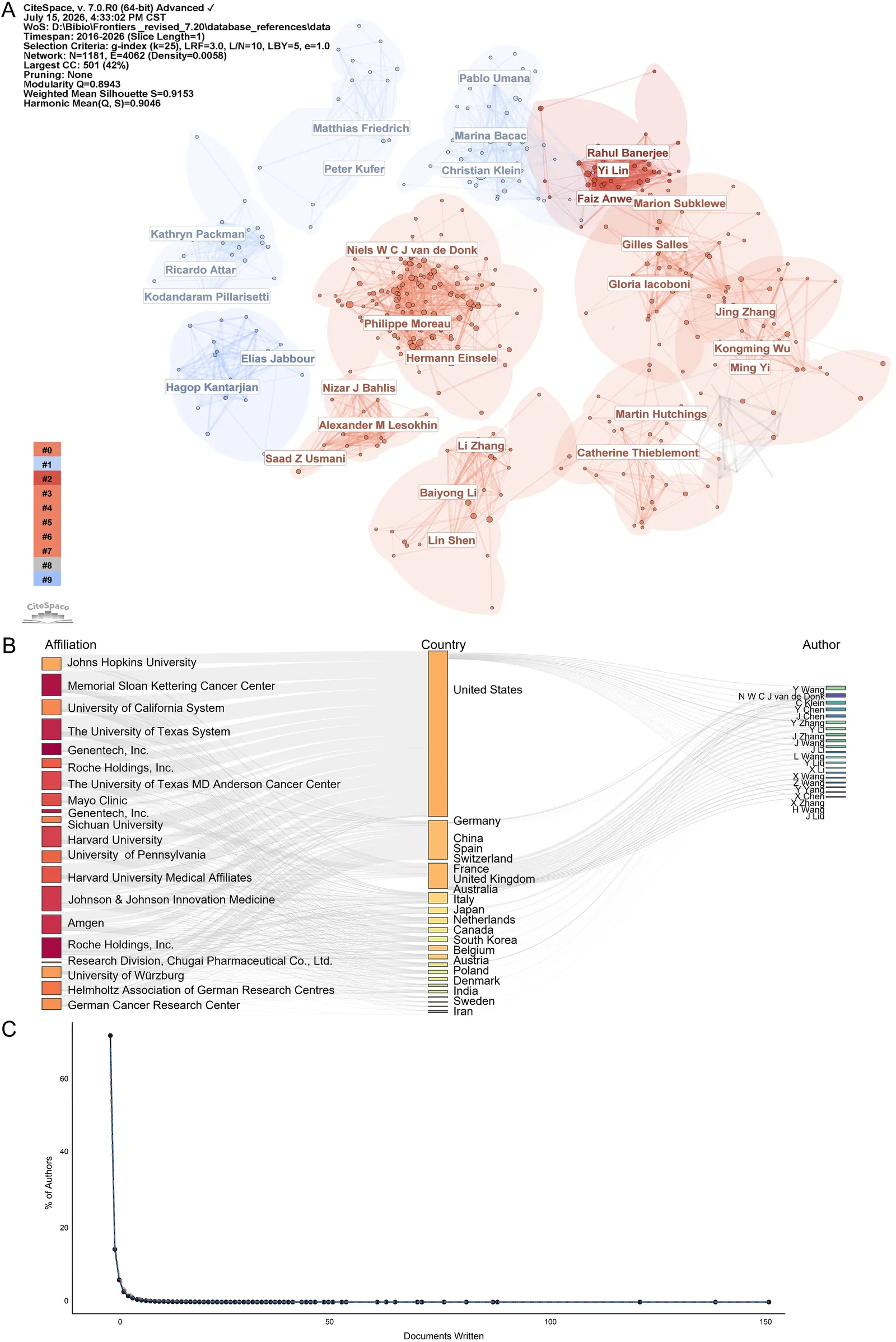
Author collaboration network and productivity analysis in bsAb research. **(A)** Authorship network cluster analysis by CiteSpace. Each node represents an author, and node size corresponds to the number of publications associated with that author. Links indicate co-authorship relationships. Colors indicate collaboration clusters identified by the clustering algorithm. **(B)** Three-field plot linking countries, institutions, and the top 20 authors. The network indicates the relationships between the top 20 authors and their countries and institutions; links represent associations between countries, institutions, and authors derived from publication affiliations. **(C)** Author productivity and Lotka’s Law generated using the R package Bibliometrix. The curve represents the relationship between the number of published articles and the corresponding number of authors. The observed distribution is compared with the theoretical Lotka distribution.

Author collaboration analysis was performed to characterize collaboration patterns, publication productivity, and citation dynamics among researchers in the bsAb field. In this analysis, we extracted author records from all collected bsAb references and identified 1,181 authorship nodes and 4,062 links using CiteSpace (**Figure 6A**). The resulting network demonstrated extensive collaboration and several densely connected author clusters, suggesting the presence of stable research communities within the bsAb field. Authorship analysis identified several major collaborative clusters, indicating that author cooperation was organized into multiple relatively independent research communities. Citation burst analysis was subsequently performed to identify authors whose publications experienced rapidly increasing scholarly attention during specific periods. The top 25 authors with the strongest citation bursts are listed (**Supplementary Figure 1**), indicating periods of rapidly increasing citation frequency to publications associated with these authors. Earlier citation bursts were mainly concentrated between 2016 and 2020, whereas several newly emerging authors exhibited burst activity beginning in 2023–2024, indicating continued evolution of the research community and sustained scholarly attention across multiple publications (**Supplementary Figure 1**). To comprehensively map interconnections across academic entities, we extracted and analyzed all the features of the literature using the R package Bibliometrix, forming an interactive network between institutions and authors. The three-field plot connected countries, institutions, and authors, illustrating the relationships among these three dimensions rather than simple publication frequency. Multiple leading authors were linked to the same institutions, suggesting that several highly productive research centers served as important hubs within the collaboration network (**Figure 6B**). The USA occupied the largest proportion of links, reflecting its broad participation across multiple institutions and research groups (**Figure 6B**). Furthermore, Lotka’s Law, formulated by Alfred James Lotka in 1926, is an empirical law that describes the distribution of productivity among authors in a scientific field (33). The relation between author productivity and publication output in bsAb research conforms to Lotka’s Law (**Figure 6C**). Approximately 67.6% of authors in this field published only one paper (**Supplementary Table 2**). In contrast, only a small proportion of authors published multiple articles, reflecting the highly skewed productivity distribution characteristic of mature scientific fields. The authorship network showed an uneven distribution of scientific productivity, with a relatively small group of highly productive or strongly connected investigators and a large proportion of authors contributing only one publication. The observed distribution was consistent with Lotka’s Law and suggested that bsAb research combines stable core research groups with broad participation from occasional contributors. Together, these analyses indicate that bsAb research is supported by a relatively small group of highly collaborative investigators alongside a large population of occasional contributors.

### 3.5 Emerging frontiers and research directions

**Figure 7:**
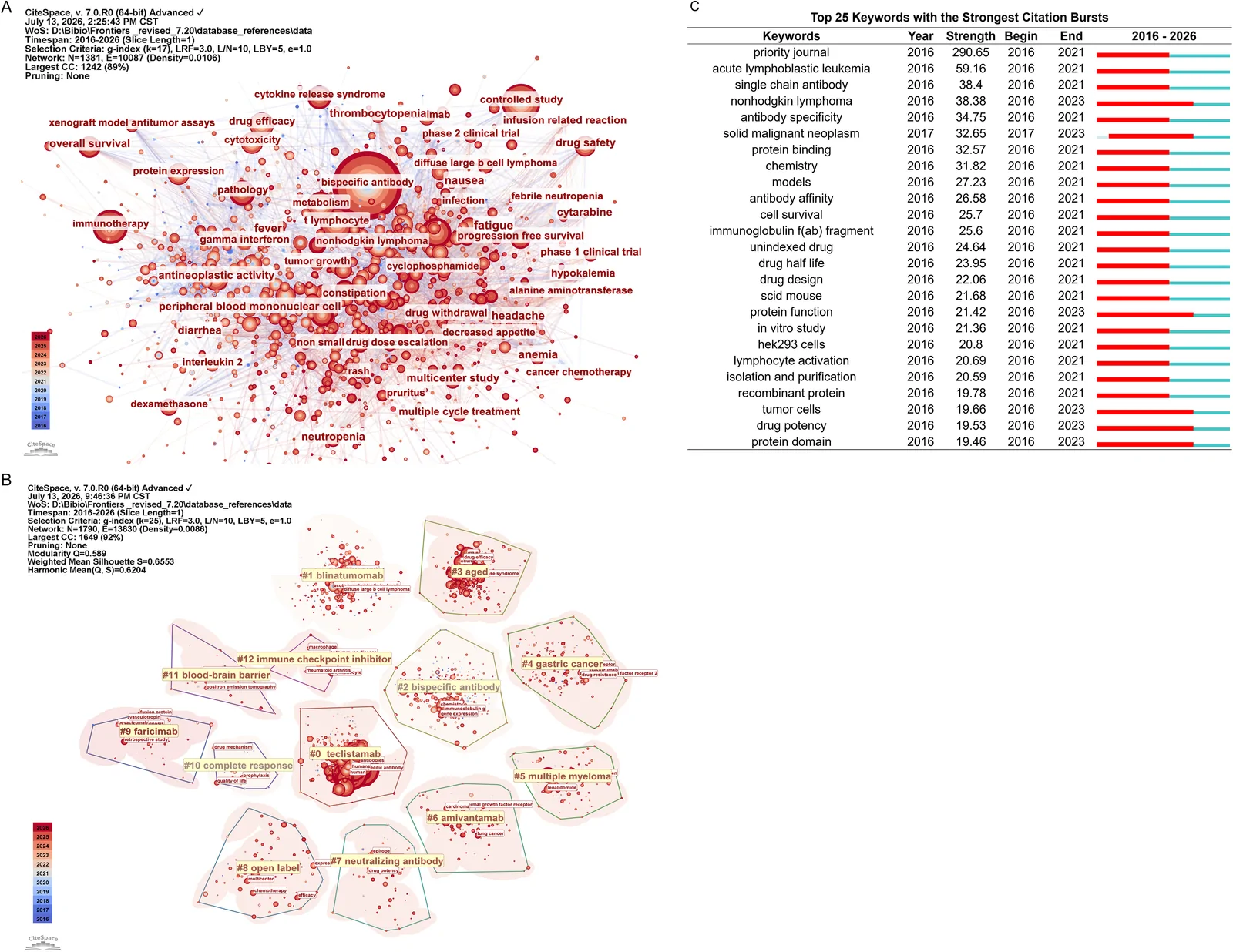
Keyword co-occurrence network, thematic clustering, and citation burst analysis of bsAb research. **(A)** Distribution of high-frequency keywords generated using CiteSpace. Different nodes indicate different keywords, and node size indicates occurrence frequency. Links represent co-occurrence relationships between keywords, and link colors indicate different years. **(B)** Clusters identified in CiteSpace, with cluster labels generated using the LLR method based on keywords. Cluster numbers are assigned according to cluster size. The automatically generated labels were interpreted together with their constituent terms and representative publications. **(C)** Keyword burst detection by CiteSpace. Burst strength represents the magnitude of a rapid increase in scholarly attention relative to the preceding period. The “keywords”, “year”, “strength”, “begin”, and “end” columns report the corresponding CiteSpace outputs. The “2016–2026” column shows the overall detection period, and red bars indicate burst duration.

**Figure 8:**
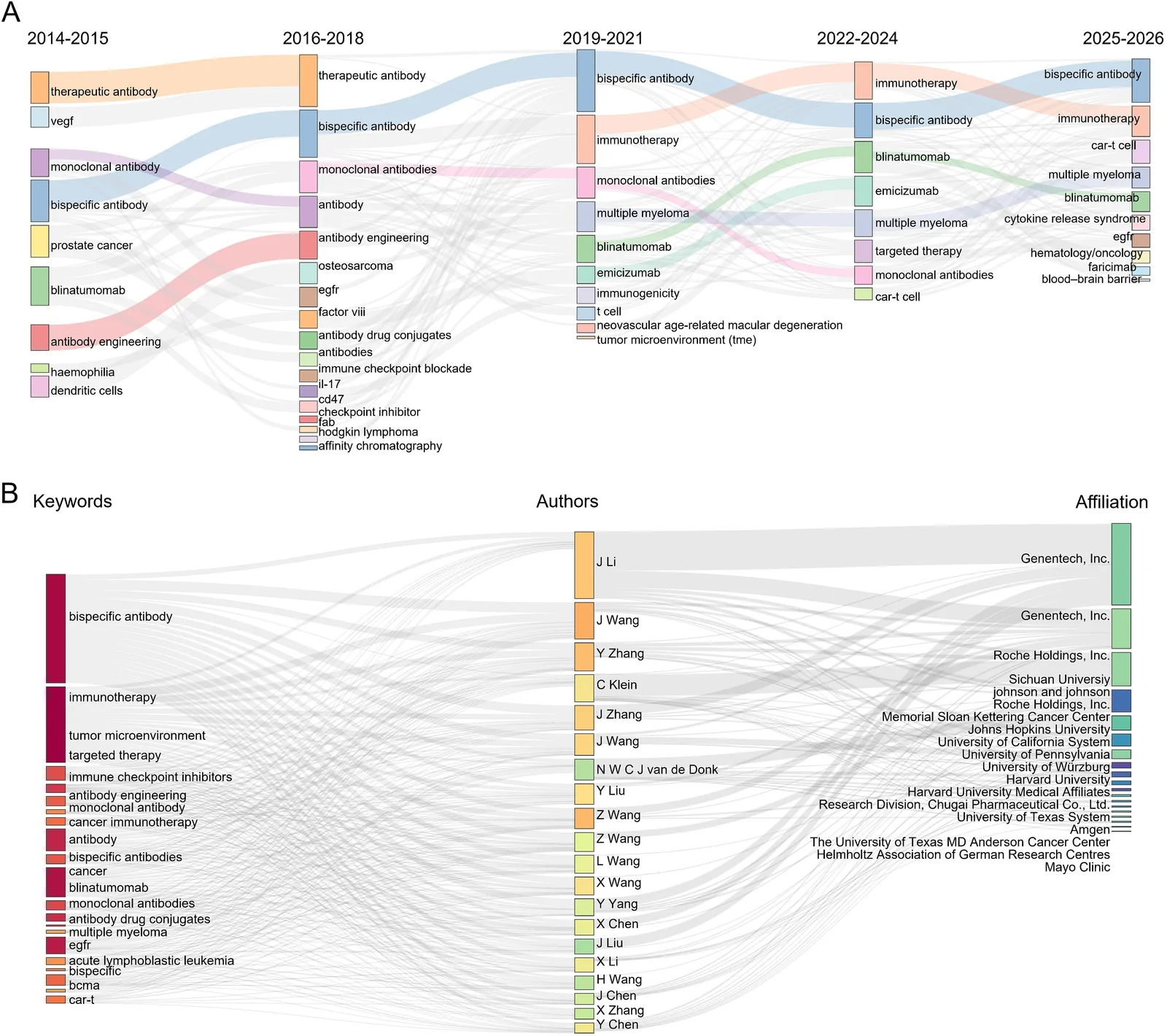
Thematic evolution and three-field analysis of research topics in bsAb studies. **(A)** Keyword evolution network across periods generated by thematic evolution analysis. Themes were identified using the Louvain clustering algorithm across five periods: 2014–2015, 2016–2018, 2019–2021, 2022–2024, and 2025–2026. Themes connected across consecutive periods indicate continuity of research focus. Node size reflects theme frequency, and connecting lines indicate shared KeyWords Plus terms between themes in successive periods. Line thickness represents the strength of thematic continuity. The map illustrates the continuation, transformation, merging, or fragmentation of research themes over time. **(B)** Three-field plot linking keywords, authors, and institutions. Line width represents the number of publications connecting institutions, authors, and keywords. The network indicates the relationship between the top 20 keywords and their authors and institutions, and crossing lines indicate the corresponding associations.

Reference citation burst analysis was performed to identify publications that experienced a rapid increase in scholarly attention during specific periods, thereby highlighting influential studies associated with different stages of bsAb development (**Supplementary Figure 2**). Most burst references were concentrated between 2010 and 2021, indicating that the conceptual foundations and early clinical development of bsAbs received sustained attention during this period. Their temporal prominence supports their interpretation as influential publications within the development of bsAbs, although burst strength reflects citation dynamics rather than comparative evidence quality. Meanwhile, CiteSpace identified 1,381 network nodes and 10,087 connections, where each node represents one keyword and node size corresponds to the occurrence frequency across the retrieved publications (**Figure 7A**). The dense keyword network suggested extensive conceptual connections among different research topics. The keywords were clustered into 13 groups and colored differently (**Figure 7B**). The identified clusters represented major thematic domains of bsAb research, including antibody engineering, hematologic malignancies, solid tumors, clinical trials, and translational immunotherapy. Keyword clusters were visualized by timeline, providing a visual representation of the evolution of research topics over time within bsAbs (**Supplementary Figure 3A**). Earlier clusters were dominated by antibody construction and molecular engineering, whereas recent clusters increasingly focused on clinical efficacy, toxicity management, and combination therapy. The time-zone view illustrates a visual representation of the temporal distribution of keywords within a specific field or discipline (**Supplementary Figure 3B**). The burst detection analysis identified the top 25 keywords with the strongest burst strength (**Figure 7C**). Citation burst analysis demonstrated a temporal transition from early engineering-related topics toward clinically oriented research themes. The temporal evolution observed in keyword bursts was broadly consistent with the burst-reference analysis, together indicating a gradual shift from antibody engineering toward clinical implementation. Additionally, the R package visualizes the cross-network evolution of keywords across years (**Figure 8A**), illustrating that early engineering-focused themes gradually evolved into disease-oriented and clinically translational research themes. All relevant keywords were divided into temporal cross-network groups. The package also showed the contribution of the top 20 keywords across networks between affiliations and authors (**Figure 8B**). In the three-field analysis, publications affiliated with Genentech and Roche-related entities were linked to a relatively large number of high-frequency keywords. The three-field analysis linked institutions, authors, and keywords, demonstrating that several highly productive institutions contributed to multiple major research themes (**Figure 8B**). The reference-burst and keyword analyses indicated a temporal evolution from foundational antibody engineering and early T-cell-engaging approaches toward clinically oriented topics involving multiple myeloma, tumor microenvironment, combination therapy, and clinical translation. Notably, the thematic-evolution map also retained several non-oncology terms, including hemophilia, factor VIII, emicizumab, neovascular age-related macular degeneration, and faricimab. These nodes were interpreted as cross-domain bibliometric carryover signals arising from shared bispecific-antibody terminology and KeyWords Plus connections, rather than as components of the oncology translational landscape. Excluding these carryover signals, the dominant oncology-oriented themes showed a progressive transition from antibody engineering and target validation toward disease-specific applications, clinical evaluation, toxicity management, and therapeutic optimization. Collectively, these analyses suggest a progressive shift from antibody engineering and target validation toward clinical translation, disease-specific applications, and optimization of therapeutic safety.

## 4 Discussion

This study provides a comprehensive bibliometric overview of global oncology-oriented bsAb research based on 5,929 publications retrieved from the Web of Science Core Collection and Scopus from 2014 to July 2026. By integrating CiteSpace, VOSviewer, and Bibliometrix, we characterized publication dynamics, collaboration networks, influential contributors, knowledge structures, and emerging research hotspots. Collectively, the findings indicate that the field has experienced sustained expansion, accompanied by increasing international collaboration and a gradual transition from antibody engineering toward clinical translation and next-generation therapeutic strategies.

Changes in cumulative citations provide a descriptive indication of scholarly attention and knowledge diffusion. The year-over-year percentage increase in cumulative citations showed substantial temporal fluctuations rather than a continuous upward trend. The rate of literature production decreased during 2015–2017, which may be attributed to the uncertain market prospects for monoclonal antibodies (34). Furthermore, the COVID-19 pandemic during 2020–2022 may have partly diverted research attention away from bsAbs (35). The slower growth in 2024–2026 may reflect four factors: renewed citation momentum, incomplete publication records for 2026, the increasing complexity of bsAb engineering, and an ongoing shift in indications toward solid tumor therapy (36). The collaboration network indicates extensive institutional connectivity, which may facilitate knowledge exchange, resource sharing, and clinical evaluation of bsAbs. Furthermore, regional differences in bsAb research activity may partly reflect differences in biomedical research capacity and policy support. According to data from the Patsnap Database (as of July 2026), 18 bsAbs had been approved for cancer treatment, 13 of which had received initial marketing authorization from the Food and Drug Administration (FDA). These approvals occurred through regulatory pathways, such as the Special Access Program for brentuximab vedotin, which includes Accelerated Approval, Breakthrough Therapy Designation, Orphan Drug Designation/Orphan Designation, and Priority Review (37). Cadonilimab, the first approved bsAb targeting PD-1 and CTLA-4, received NMPA special regulatory designations, including Breakthrough Therapy Designation, Conditional Approval, and Priority Review (37, 38). These bibliometric patterns are consistent with substantial international research connectivity in the bsAb field, but they do not establish that collaboration directly caused clinical or regulatory outcomes.

Within the collaboration network, Rigshospitalet and Genentech were linked to research involving a phase 1 trial of glofitamab in patients with relapsed or refractory B-cell non-Hodgkin lymphoma, in which a complete response rate of 53% was reported (39, 40). Johnson & Johnson Innovative Medicine also completed a phase 3 trial of amivantamab plus chemotherapy in patients with advanced non-small-cell lung cancer (NSCLC) harboring epidermal growth factor receptor (EGFR) exon 20 insertions; the 18-month progression-free survival rate was 31% (41, 42). This trial involved multiple clinical centers, including Tongji University School of Medicine and the First Affiliated Hospital of Sun Yat-sen University in China, Yonsei University College of Medicine in South Korea, and Vall d’Hebron University Hospital in Spain, among others (41, 42). This example illustrates the multicenter and international nature of clinical evaluation in the bsAb field and is presented as clinical context for the collaboration network.

Over the past decade, institutional collaboration may have supported resource integration and coordination during drug development; however, bibliometric data alone cannot establish this effect. Institutional burst detection identifies organizations that received rapidly increasing citation attention during specific periods. Clinical trial findings published in influential journals may inform regulatory evaluation and clinical decision-making. For instance, clinical trial findings on teclistamab published in The Lancet and The New England Journal of Medicine for patients with relapsed or refractory multiple myeloma achieved an overall response rate of 63% and 11.3-month progression-free survival, and its manageable safety profile supported the European Medicines Agency (EMA) approval of teclistamab (39, 43, 44). Highly cited author clusters may delineate influential research streams that have contributed to the development of clinical evidence and therapeutic concepts (45, 46). The cluster related to van de Donk, Moreau, and Einsele has contributed to clinical trials in patients with relapsed/refractory multiple myeloma, in which subcutaneous talquetamab demonstrated high and durable response rates (overall response rate 67%–74%). Indirect comparisons using real-world external controls, talquetamab significantly improved depth of response (relative response ratio for ≥ complete response as high as 52.22), progression-free survival (hazard ratio 0.30–0.47), and overall survival (hazard ratio, 0.35–0.37) (47, 48).

The thematic-evolution analysis showed that the earlier literature was dominated by broad concepts related to therapeutic antibody research, including antibody engineering, affinity, specificity, and early disease-focused applications. A small number of non-oncology terms visible in the map, including haemophilia, factor VIII, Emicizumab, neovascular age-related macular degeneration, and faricimab, were treated as cross-domain bibliometric carryover signals and were excluded from the clinical interpretation of the oncology-oriented thematic landscape. From 2019 onward, “bispecific antibody” and “immunotherapy” became persistent central themes and were increasingly connected to named agents, defined molecular targets, specific diseases, clinical-trial phases, efficacy outcomes, and treatment-related toxicities. During 2022–2026, mature hematologic applications, including blinatumomab, multiple myeloma, and T-cell redirection, coexisted with newer topics such as EGFR-directed therapy, cytokine-release syndrome (CRS), acquired resistance, and tissue-specific delivery. This pattern suggests an expansion, rather than a simple replacement, of research priorities: platform engineering remains foundational, whereas the research focus has increasingly shifted toward clinical implementation, toxicity management, resistance, and indication-specific optimization. The clinical trials, agents, and platforms discussed below were selected because they corresponded to prominent keyword clusters, thematic-evolution pathways, highly cited references, references with strong citation bursts, or other identifiable signals in the bibliometric dataset. They are used to contextualize the quantitative findings rather than provide a formal comparative synthesis of clinical efficacy, safety, or treatment outcomes.

The strongest historical signal was the development of CD19 × CD3 T-cell engagement in acute lymphoblastic leukemia (ALL). The large keyword cluster centered on blinatumomab and ALL was accompanied by the two strongest reference bursts in the dataset: the randomized study by Kantarjian et al. (burst strength, 90.01) and the phase 2 study by Topp et al. (77.30). In the multicenter phase 2 study, blinatumomab produced clinically meaningful responses in adults with relapsed or refractory B-precursor ALL (49). The subsequent randomized phase 3 trial showed longer median overall survival with blinatumomab than with standard chemotherapy (7.7 months *vs*. 4.0 months), providing clinical evidence that T-cell-engaging bsAbs could improve a clinically meaningful survival endpoint in this setting (10). The prominence of measurable residual disease in this research stream is consistent with evidence that blinatumomab can eradicate residual leukemia in a substantial proportion of patients (50). Taken together, these bibliometric and clinical signals illustrate an important translational sequence from immune-redirection concepts to multicenter clinical evaluation, survival outcomes, and disease-monitoring applications. However, the prominence of this cluster should be interpreted as evidence of historical influence and clinical maturity within the literature, not as proof that blinatumomab is superior to all subsequent bsAb strategies.

The more recent literature is dominated by B-cell malignancies and multiple myeloma. This interpretation is supported by the multiple-myeloma/B-cell maturation antigen (BCMA) cluster, the persistent appearance of multiple myeloma in the thematic-evolution map, and the ongoing 2024–2026 citation bursts of studies evaluating teclistamab, talquetamab, elranatamab, glofitamab, and epcoritamab. In large B-cell lymphoma, fixed-duration glofitamab and subcutaneous epcoritamab produced substantial complete-response rates in heavily pretreated populations, including patients previously exposed to chimeric antigen receptor T-cell (CAR-T) therapy (51, 52). In multiple myeloma, teclistamab established BCMA × CD3 redirection as an active off-the-shelf approach, whereas talquetamab and elranatamab extended the field toward GPRC5D and additional BCMA-directed formats (44, 53, 54). These studies help explain why “bispecific antibody”, “multiple myeloma”, “phase 1 clinical trial”, “phase 2 clinical trial”, “complete response”, and “progression-free survival” occupy central positions in the updated co-occurrence network. The concurrent emergence of CAR-T cells is best interpreted as intellectual and clinical adjacency rather than as inclusion of CAR-T therapy within the bsAb category: the two modalities share targets, immune-effector mechanisms, toxicity frameworks, and sequencing questions and are therefore frequently discussed and cited together. Importantly, publication frequency and citation prominence reflect scientific attention and influence, but do not by themselves establish comparative efficacy, durability, commercial maturity, or therapeutic superiority.

The updated network further suggests that the field is moving from asking whether bsAbs are active to determining how they can be administered safely, reproducibly, and at scale. CRS, infections, infusion-related reactions, neutropenia, febrile neutropenia, anemia, thrombocytopenia, fatigue, treatment discontinuation, dose escalation, and multicenter or controlled studies were all prominent in the co-occurrence map. Clusters labeled “aged”, “open label”, and “complete response” are therefore better understood as demographic, study-design, and outcome dimensions of clinical maturation than as independent mechanistic themes. This interpretation is supported by the clinical literature. In MajesTEC-1, CRS occurred in 72.1% of teclistamab-treated patients but was predominantly grade 1–2 and clustered around step-up dosing; protocolized monitoring and timely use of tocilizumab were associated with fewer recurrent events (55). With longer follow-up, infections occurred in 80.0% of patients and grade 3–4 infections in 55.2%, indicating that prolonged immune dysfunction may become at least as clinically consequential as the typically early and manageable CRS (56). Epcoritamab similarly showed frequent but predominantly low-grade CRS, together with a smaller risk of immune effector cell-associated neurotoxicity syndrome (52). These findings support greater emphasis on infection prophylaxis, immunoglobulin monitoring or replacement, vaccination, outpatient step-up strategies, frailty and age-specific assessment, standardized reporting of CRS and neurotoxicity, and long-term follow-up. They also highlight the logistical burden of CD3-engager therapy, including observation requirements, rapid access to experienced clinical teams, and the resources needed to manage acute immune-mediated toxicities.

Target diversification and treatment resistance formed another emerging research axis. The persistence of BCMA-directed agents and the rise of G protein-coupled receptor class C group 5 member D (GPRC5D)-directed therapy indicate that the field is no longer centered on a single target. At the same time, the increasing prominence of “progression”, “drug efficacy”, “tumor growth,” and related terms is consistent with growing attention to the incomplete durability of T-cell-redirecting therapy. Genomic analysis of patients relapsing after BCMA- or GPRC5D-directed CAR-T cell or T-cell engager therapy identified biallelic target loss and extracellular-domain alterations that impaired antibody binding, demonstrating that antigen escape can be selected under therapeutic pressure (57). These observations provide a biological context for the bibliometric transition from general antibody engineering toward target selection, treatment sequencing, combination therapy, and acquired resistance. Future studies should therefore incorporate longitudinal assessment of target expression, biomarkers of T-cell fitness and exhaustion, rational sequencing of BCMA- and GPRC5D-directed modalities, and molecular designs capable of retaining activity in the presence of heterogeneous or evolving antigen expression. Publication growth in these areas should nevertheless be regarded as an indicator of increasing research activity rather than direct evidence that the underlying resistance problems have been resolved.

Although hematologic malignancies remain the most clinically mature area, the cluster structure indicates continued expansion into solid tumors. One sizeable cluster was characterized by gastric cancer, breast cancer, HER2, and prostate cancer, whereas another cohesive cluster centered on amivantamab, EGFR, and NSCLC. The phase 3 PAPILLON trial showed that adding the EGFR × MET bsAb amivantamab to chemotherapy improved progression-free survival in untreated advanced NSCLC with EGFR exon 20 insertions (41). This trial provides a clinically relevant counterpart to the appearance of EGFR and targeted therapy in the recent thematic map. Nevertheless, the solid-tumor clusters remained more heterogeneous and less dominant than the hematologic clusters, consistent with the additional biological and pharmacological barriers encountered in solid tumors. These include heterogeneous or unstable target expression, limited penetration into tumor tissue, an immunosuppressive tumor microenvironment, impaired T-cell fitness, compensatory signaling, and on-target/off-tumor toxicity caused by low-level antigen expression in normal tissues. Consequently, progress in solid tumors is likely to depend not only on identifying paired targets but also on optimizing valency, affinity, conditional activation, pharmacokinetics, spatial selectivity, biomarker-guided patient selection, and rational combinations. The increasing visibility of solid-tumor terms therefore indicates an active area of investigation, but should not be equated with the level of clinical consolidation already achieved in several hematologic malignancies.

Compared with previous reviews focusing primarily on therapeutic mechanisms or clinical outcomes, this study integrated two major bibliographic databases and multiple complementary bibliometric platforms to provide a comprehensive knowledge map of oncology-oriented bsAb research. The combined use of CiteSpace, VOSviewer, and Bibliometrix enabled cross-validation of collaboration patterns, thematic evolution, and citation dynamics, thereby improving the robustness and interpretability of the findings.

Several limitations should be considered when interpreting these findings. First, the analysis was limited to English-language articles and reviews indexed in the Web of Science Core Collection and Scopus and was constrained by predefined subject categories and search terminology. Although the use of two databases broadened literature coverage, relevant studies indexed exclusively under platform names, individual drug names, or alternative terms, such as BiTE, DART, dual-affinity retargeting, or T-cell redirecting antibodies, may not have been captured. These databases also predominantly index peer-reviewed academic publications and may incompletely represent clinical trial registries, regulatory documents, conference abstracts, patents, industry reports, unpublished studies, and studies reporting negative results. Consequently, clinically important products with limited journal publication output may be underrepresented.

Second, cross-database deduplication, author-name disambiguation, and normalization of country, institution, company, journal, and keyword names reduced but could not eliminate inconsistencies in database indexing. Residual inconsistencies may divide a single entity into multiple nodes or combine distinct entities, thereby affecting estimates of productivity, collaboration, and centrality. Country-level findings may also be influenced by multinational authorship, multiple institutional affiliations, and the affiliation-counting method used. The inherent limitations of the R package Bibliometrix do not allow institutions to be merged after standardization.

Third, citation-based indicators are affected by citation lag, self-citation, field-specific citation practices, and publication bias. Self-citations were not excluded or adjusted for separately and may therefore have influenced the citation rankings and network structure. In addition, the 2026 observation period was incomplete because the final search was conducted on 8 July 2026. Publication growth estimates and thematic trends for the most recent period should therefore be interpreted cautiously, as recent publications and research topics have had less time to accumulate citations or generate stable burst signals.

Fourth, automatically generated cluster labels may represent demographic, methodological, or outcome-related descriptors, such as “aged” or “open label”, rather than discrete biological or clinical themes. These labels therefore require interpretation in conjunction with their constituent terms, network positions, and representative publications. As the thematic-evolution analysis incorporated KeyWords Plus terms, cross-domain terminology may persist despite oncology-oriented database filtering. Accordingly, non-oncology nodes were retained for transparency but were not interpreted as oncology-related translational themes.

Finally, bibliometric methods identify patterns of association, visibility, citation influence, and scientific connectivity but cannot establish causal relationships among national policies, institutional collaboration, publication growth, and translational or regulatory outcomes. Publication and citation measures should therefore not be interpreted as direct indicators of therapeutic superiority, clinical maturity, commercial success, or innovation. Moreover, the qualitative clinical discussion presented in this study is a narrative synthesis intended to contextualize the bibliometric findings and does not constitute a formal systematic review of efficacy, safety, or comparative clinical evidence.

## 5 Conclusion

This bibliometric study provides a comprehensive overview of the global research landscape of oncology-oriented bsAbs. Based on 5,929 publications, the analysis identified sustained growth in scientific output, increasing international collaboration, and evolving research priorities from antibody engineering toward clinical translation, combination immunotherapy, and novel therapeutic targets. The findings highlight influential contributors, major knowledge domains, and emerging research frontiers while emphasizing that bibliometric associations should not be interpreted as causal evidence of clinical success. Overall, this study offers a bibliometric knowledge framework that may support future research planning, interdisciplinary collaboration, and the strategic development of next-generation bsAbs for cancer therapy.

## Data Availability

The raw data supporting the conclusions of this article will be made available by the authors, without undue reservation.
