# Supplementary material for: Global development of bispecific antibodies in oncology: a bibliometric analysis of geographic disparities, collaborative models, and emerging translational strategies
Source: Front Immunol. 2026 Aug 20;17:1884087. doi: 10.3389/fimmu.2026.1884087 (PMC13538842; doi:10.3389/fimmu.2026.1884087)
Supplement: Supplementary file 1 [file DataSheet1.docx]

**Supplementary Information**

Global Development of Bispecific Antibodies in Oncology: A Bibliometric Analysis of Geographic Disparities, Collaborative Models, and Emerging Translational Strategies

# 1 Supplementary tables and figures

**Table S1.** Top 10 journals in zone 1 (from R package Bibliometrix).

| Journal | Rank | Publications | Cumulative publications | Zone |
| --- | --- | --- | --- | --- |
| Frontiers in Immunology | 1 | 254 | 254 | Zone 1 |
| Mabs | 2 | 206 | 460 | Zone 1 |
| Cancers | 3 | 168 | 628 | Zone 1 |
| Journal for Immunotherapy of Cancer | 4 | 132 | 760 | Zone 1 |
| Frontiers in Oncology | 5 | 105 | 865 | Zone 1 |
| Blood | 6 | 102 | 967 | Zone 1 |
| Journal of Clinical Oncology | 7 | 100 | 1067 | Zone 1 |
| International journal of molecular sciences | 8 | 90 | 1157 | Zone 1 |
| Blood Advances | 9 | 75 | 1232 | Zone 1 |
| Clinical Cancer Research | 10 | 67 | 1299 | Zone 1 |

* Cumulative publications are the sum of corresponding frequency and all frequency of higher ranks.

**Figure S1.** Citation burst analysis of authors generated by CiteSpace. Top 25 authors sorted by the beginning year of burst. Author burst detection by CiteSpace. Institutions are ranked according to burst strength. "Year" column indicates the year detected; "Strength" column represents the burst intensity; "Begin" and "End" columns indicate the start and end times of the burst. "2016–2026" column represents the time range of detection, where the red line represents the time range of burst duration. Citation bursts identify periods during which an author's publications experienced rapidly increasing citation attention and do not directly measure overall scientific impact or publication productivity.

**Table S2.** Author productivity through Lotka's Law (from R package “Bibliometrix”).

| Document written | Number of Authors | Proportion of Authors % | Theoretical % |
| --- | --- | --- | --- |
| 1 | 20134 | 71.21 | 67.56 |
| 2 | 3985 | 14.09 | 14.49 |
| 3 | 1669 | 5.90 | 5.88 |
| 4 | 780 | 2.76 | 3.1 |
| 5 | 460 | 1.63 | 1.9 |
| 6 | 302 | 1.07 | 1.26 |
| 7 | 192 | 0.68 | 0.9 |
| 8 | 135 | 0.48 | 0.67 |
| 9 | 105 | 0.37 | 0.51 |
| 10 | 81 | 0.29 | 0.41 |

* Proportion of author = number of author/sums of all number of authors *100%.

* Theoretical is theoretical value of number of authors from Lotka's Law.

**Figure S2.** References burst analysis by CiteSpace. Top 25 references sorted by the beginning year of burst. " References " column indicates the references with the strongest burst detection in the top25. "Year" column indicates the year detected; "Strength" column represents the burst intensity; "Begin" and "End" columns indicate the start and end times of the burst. "2016–2026" column represents the time range of detection, where the red line represents the time range of burst duration.

**Figure S3.** Keywords cluster distribution. A. Keywords cluster analysis in time line by CiteSpace. Each keyword is positioned along a timeline, with the x-axis representing time and the y-axis representing the frequency or importance of the keyword. B. Keywords cluster analysis in time zone by CiteSpace. Keyword cluster distribution over a time interval.

| **Table S3.** Parameter settings for bibliometric analysis software. | | | | | |
| --- | --- | --- | --- | --- | --- |
| **Software and version** | **Analysis module** | **Data scope / term source** | | **Thresholds / limits** | **Normalization / clustering / model** |
| VOSviewer 1.6.20 | Country citation network | Combined deduplicated WoSCC and Scopus dataset; 197 countries identified. | | Minimum 90 publications per country; 22 countries retained. | Association-strength normalization. |
|  | Source co-citation network | 1,014 cited sources identified in the combined dataset. | | Minimum 15 citations per cited source; 83 cited sources retained. | Association-strength normalization; VOSviewer clustering of closely related cited sources. |
| CiteSpace 7.0.R0 Advanced | Common temporal and node-selection settings | Study period: 2014 to July 8, 2026; time slice: 1 year. Term sources for term-based analyses: title, abstract, author keywords, and Keywords Plus. | | g-index scale factor k = 25; No network pruning applied. | Modified g-index node-selection procedure; Cosine similarity for similarity/normalization： |
|  | Country collaboration network | Country nodes derived from author-address metadata across the full study period. | | Country frequency ≥ 80 | CiteSpace network layout; betweenness centrality calculated as described below. |
|  | Institution collaboration network | Institutional affiliations extracted from the full bibliographic dataset. | | Institution frequency ≥ 100 | CiteSpace network layout. |
|  | Author collaboration network | Authors extracted from the full bibliographic dataset. | | Betweenness ≥ 3 | Clusters generated from the author collaboration networ using the log-likelihood ratio (LLR) method. |
|  | Keyword co-occurrence and clustering | Terms extracted from titles, abstracts, author keywords, and Keywords Plus. | | Keyword occurrence frequency ≥ 30 for the reported visualization or summary. | Clusters generated from the keyword co-occurrence network; labels extracted from keywords using the log-likelihood ratio (LLR) method. |
| **Table S3.** (Continued) | | | | | |
|  | Reference co-citation and clustering | | Cited references from the combined dataset across 2014–2026. | No additional frequency threshold specified beyond the annual g-index selection. | Co-citation clustering; cluster labels generated using the LLR method based on keywords associated with the cluster. |
|  | Burst detection | | Applied over annual slices from 2014 to July 8, 2026. | Reported burst strength ≥ 3; minimum burst duration = 3 years. | Number of states = 2; state-intensity ratio αᵢ/αᵢ₋₁ = 2.0; transition-cost parameter γ = 1.0. |
|  | Betweenness centrality and cluster-quality reporting | | Applied to applicable CiteSpace networks. | Nodes with betweenness centrality > 0.10 highlighted. | Purple outer ring = node exceeding the centrality threshold. |
| Bibliometrix 5.0 in R 4.5.1 | General bibliometric performance analysis | | Deduplicated and normalized WoSCC and Scopus records merged into one bibliographic file. | No additional analytical threshold specified. | Package functions for annual production/citation trends, country contribution, institutional productivity, author productivity, source distribution, and keyword evolution. |
|  | Bradford and Lotka analyses | | Sources and author productivity calculated from the merged bibliographic dataset. | No visualization threshold specified. | Bradford zones and fitted Lotka power-law distribution. |
|  | Three-field plots | | Fields selected according to the specific figure. | Top 20 most frequent items in each relevant field. | Frequency-based display limit. |
|  | Thematic evolution | | Conceptual field: Keywords Plus. Time intervals: 2014–2018, 2019–2021, 2022–2024, and 2025–2026. | Maximum 600 terms per period; minimum cluster frequency = 1. | Louvain community-detection algorithm based on modularity optimization. |
